# Bronchosubcutaneous fistula manifesting as massive subcutaneous emphysema treated successfully by endobronchial Watanabe spigots

**DOI:** 10.1002/rcr2.652

**Published:** 2020-08-24

**Authors:** Masahiro Yanagiya, Noriko Hiyama, Hideyuki Takeshima, Kazuhiro Usui, Jun Matsumoto

**Affiliations:** ^1^ Department of General Thoracic Surgery NTT Medical Center Tokyo Tokyo Japan; ^2^ Division of Respirology NTT Medical Center Tokyo Tokyo Japan

**Keywords:** Bronchial occlusion, bronchosubcutaneous fistula, endobronchial Watanabe spigot, subcutaneous emphysema, virtual bronchoscopic navigation

## Abstract

A bronchosubcutaneous fistula (BF) is an abnormal communication between the bronchus, pleural cavity, and subcutaneous tissue. Treatment of BF has been documented rarely. We describe a successful endoscopic bronchial occlusion using endobronchial Watanabe spigots (EWSs) for BF manifesting as massive subcutaneous emphysema (SE). A 78‐year‐old woman developed delayed localized SE following a surgical pleural biopsy for the diagnosis of primary lung cancer. Computed tomography (CT) of the chest revealed BFs resulting from pleural biopsy. The affected bronchi were identified using chest CT. We inserted EWSs into the affected bronchi with the aid of virtual bronchoscopic navigation. This bronchoscopic procedure achieved complete resolution of the SE by sealing the BFs without the need for surgical interventions.

## Graphical abstract

In this report, we describe a successful endoscopic bronchial occlusion using endobronchial Watanabe spigots for bronchosubcutaneous fistula manifesting as massive subcutaneous emphysema.

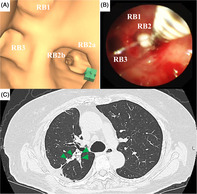

## Introduction

A bronchosubcutaneous fistula (BF; also known as a bronchopleural subcutaneous fistula) is a rare (but problematic) condition characterized by an abnormal communication between the bronchus, pleural space, and subcutaneous tissue [1]. As BF has been rarely documented, its frequency still remains unknown [1, 2]. BF presents with subcutaneous emphysema (SE) and can cause respiratory symptoms [1, 2].

A few reports described surgical interventions such as incisional drainage of SE [2].

We describe a successful treatment of BFs via placement of endobronchial Watanabe spigots (EWSs) without surgical intervention.

## Case Report

A 78‐year‐old woman with a history of hypertension and no history of smoking complained of a sustained cough. She visited our hospital, and was diagnosed initially as having pneumonia. Although her symptoms improved, a follow‐up chest X‐ray revealed a small right‐sided pleural effusion that appeared inconsistent with pneumonia. Computed tomography (CT) of the chest revealed multiple pleural nodules and an irregular 17‐mm pulmonary nodule located in the right upper lobe in addition to the pleural effusion. The serum level of carcinoembryonic antigen was increased at 75.7 ng/mL (reference range < 3.4 ng/mL). Positron emission tomography and brain magnetic resonance imaging excluded extrathoracic distant metastases. A pleural effusion owing to primary lung cancer was suspected. To obtain a pathological specimen, we conducted a surgical biopsy of right pleura via mini‐thoracotomy under general anaesthesia. Although we obtained adequate pleural samples for pathology, we injured a part of the lung surface intraoperatively owing to tight adhesions between parietal pleura and the lung surface. We applied a polyglycolic‐acid sheet and fibrin glue to fix the injured area. The patient was discharged with no complications. The pathological diagnosis was pleuritis owing to primary lung adenocarcinoma, pT3N0M1a stage IVa (eighth TNM classification). A mutation on an epidermal growth factor receptor gene with an exon‐19 deletion was detected in pathological tissue. She began treatment with osimertinib three weeks after biopsy.

**Figure 1:**
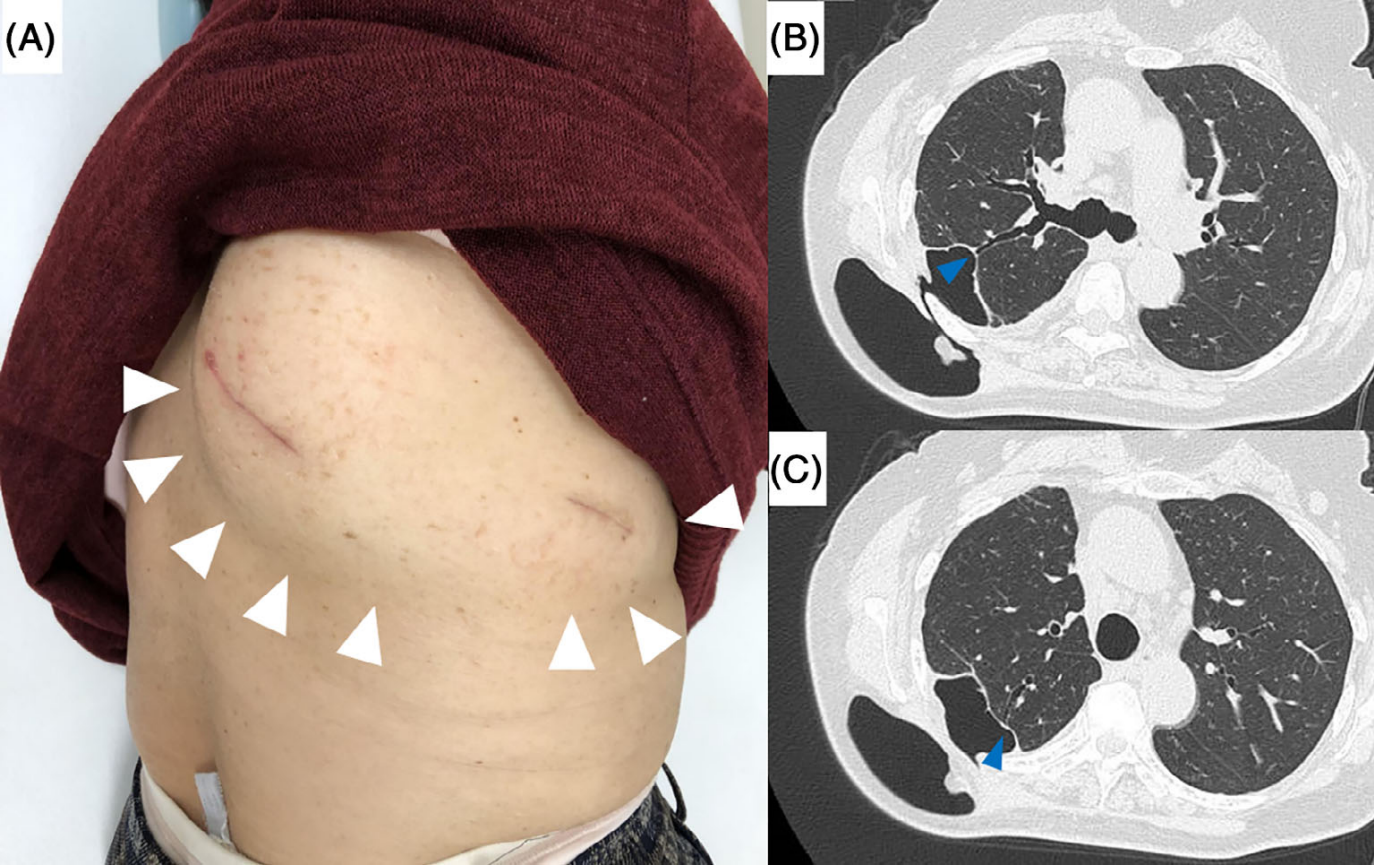
(A) Massive subcutaneous emphysema (SE) along the incisions of the right chest (white arrows). (B, C) Computed tomography of the chest showed SE and abnormal communications between subsegmental bronchi (blue arrows; (B) RB2b; (C) RB2a), pleural space, and subcutaneous tissue.

One month after surgery, she noticed SE along the incisions of the right chest. SE was localized and small initially, so we continued treatment with osimertinib without interventions. However, SE gradually progressed. Three months after surgery, she presented with massive right SE (Fig. 1A) and complained of pain. CT image of the chest revealed right BFs (Fig. 1B, C). Because pleural biopsy had injured a corresponding part of the pleura and the primary tumour effectively shrunk on treatment of osimertinib, BF resulted presumably from both pleural biopsy and osimertinib. CT suggested subsegmental bronchi responsible for the BFs, so endoscopic bronchial occlusion was indicated to repair them.

**Figure 2:**
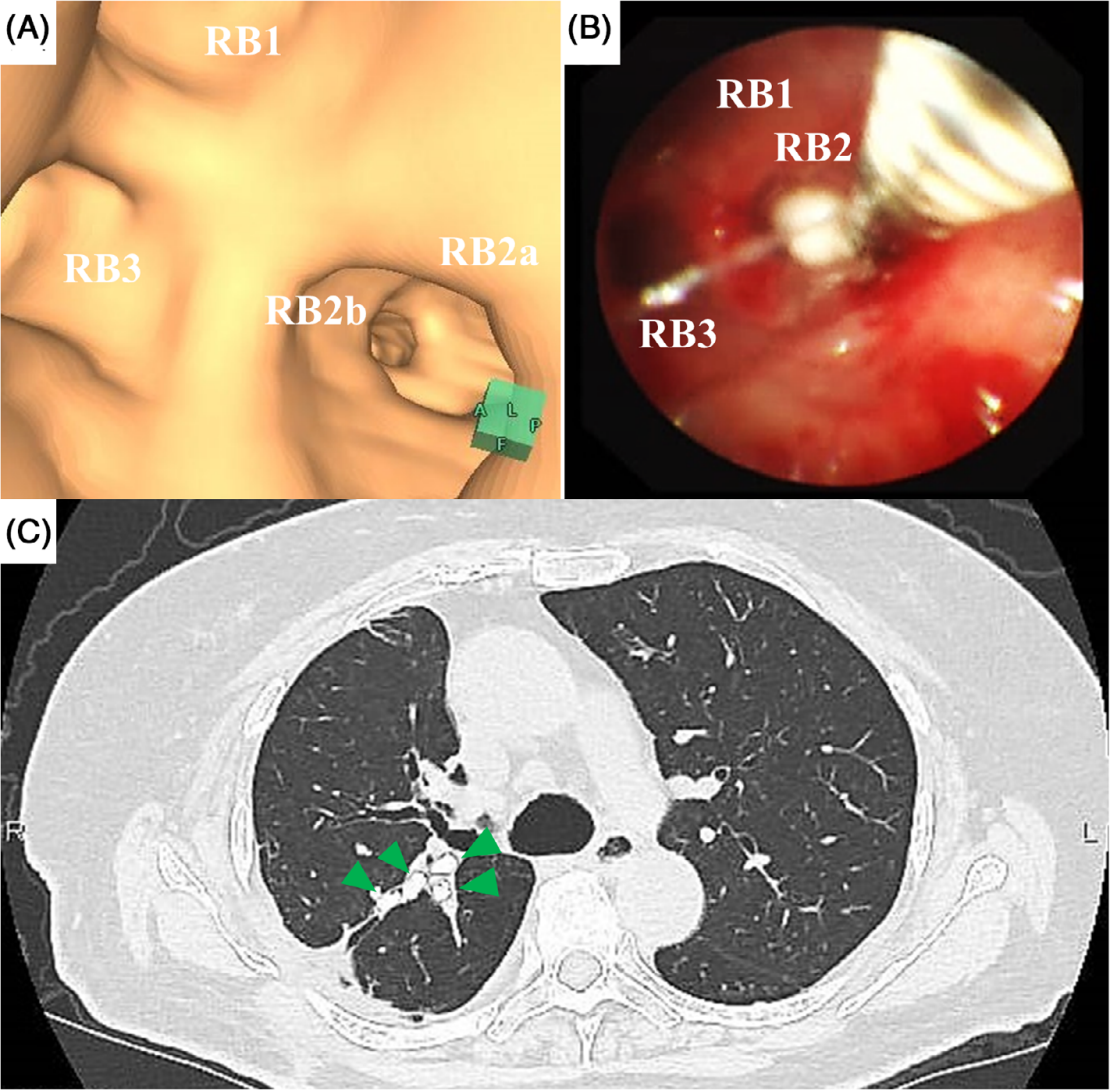
(A) Image by virtual bronchoscopic navigation of affected subsegmental bronchi. RB2b and RB2a were responsible for bronchosubcutaneous fistulae. (B) Bronchoscopic image showing insertion of an endobronchial Watanabe spigot (EWS) into RB2b. (C) Computed tomography image of the chest after the bronchoscopic procedure showed appropriate deployment of EWSs (two at RB2b, one at RB2a, and one at the root of RB2) (green arrows) and complete disappearance of subcutaneous emphysema.

Using the plain CT image and the virtual bronchoscopy navigation (VBN) image reconstructed by CT, RB2b and RB2a were identified as the affected subsegmental bronchi responsible for SE (Figs. 1B, C, 2A). Two weeks after observing massive SE, we undertook bronchial occlusion using EWSs (Novatech; Cedex, France) under general anaesthesia. After endotracheal intubation, we used a flexible bronchoscope (BF‐1T‐260; Olympus, Japan) and V‐shaped grasping forceps (FG‐14P‐1; Olympus) to insert EWSs into the affected airways (Fig. 2B). We inserted one 5‐mm EWS into RB2bi, one 5‐mm EWS into the stem of RB2b, and one 6‐mm EWS into RB2a. Finally, an additional 6‐mm EWS was deployed at the root of RB2. She recovered well and was discharged with no complications. Two weeks later, the SE had completely resolved. CT of the chest at one month showed appropriate deployment of EWSs (Fig. 2C). At four months, there was no recurrence of SE and no complications related to the EWS. After six months of therapy with osimertinib, there was no tumour progression.

## Discussion

As bronchial occlusion was a well‐known treatment for bronchopleural fistula [3, 4, 5], the management of a BF with EWS was an extension of current practice. Although we were unsure that SE could be treated with bronchial occlusion only, SE improved dramatically without surgical intervention. She did not develop a subcutaneous abscess and, thus, avoided therapeutic surgical drainage.

An EWS is a bronchial spigot made of silicon used to treat various disorders related to bronchial or pulmonary fistulae [3]. Besides EWSs, endobronchial occlusion can also be achieved by cyanoacrylate, a polymer glue sealant, or endobronchial valves [4, 5]. In our patient, the diameter of target subsegmental bronchi was relatively large (6 mm). Considering availability and the size of target BFs, we selected EWSs to achieve bronchial occlusion.

VBN contributed greatly to the success of our procedure in two ways. The first was accurate localization of the bronchi responsible for the BFs [5]. Conventionally, determination of affected bronchi necessitates the balloon occlusion test [3]. However, with the aid of VBN in addition to the plain CT image, we identified the points of the BFs and affected bronchi. In our case, the balloon occlusion test would have been challenging without therapeutic drainage, so identifying the affected bronchi accurately by VBN was important. The second way was simulation of the bronchoscopic procedure. On the basis of the VBN image, we obtained a vivid image of the route to the target bronchi. VBN demonstrates the virtual endobronchial view of the airway non‐invasively, and facilitates the endobronchial procedure effectively [5].

In conclusion, we undertook endobronchial occlusion for BFs successfully. EWSs and VBN were helpful in this procedure.

### Disclosure Statement

Appropriate written informed consent was obtained for publication of this case report and accompanying images.
